# Key Technology of Real-Time Road Navigation Method Based on Intelligent Data Research

**DOI:** 10.1155/2016/1874945

**Published:** 2016-10-31

**Authors:** Haijing Tang, Yu Liang, Zhongnan Huang, Taoyi Wang, Lin He, Yicong Du, Xu Yang, Gangyi Ding

**Affiliations:** School of Software, Beijing Institute of Technology, Beijing 100081, China

## Abstract

The effect of traffic flow prediction plays an important role in routing selection. Traditional traffic flow forecasting methods mainly include linear, nonlinear, neural network, and Time Series Analysis method. However, all of them have some shortcomings. This paper analyzes the existing algorithms on traffic flow prediction and characteristics of city traffic flow and proposes a road traffic flow prediction method based on transfer probability. This method first analyzes the transfer probability of upstream of the target road and then makes the prediction of the traffic flow at the next time by using the traffic flow equation. Newton Interior-Point Method is used to obtain the optimal value of parameters. Finally, it uses the proposed model to predict the traffic flow at the next time. By comparing the existing prediction methods, the proposed model has proven to have good performance. It can fast get the optimal value of parameters faster and has higher prediction accuracy, which can be used to make real-time traffic flow prediction.

## 1. Introduction

Traditional traffic flow forecasting methods supported by Intelligent Transportation System have gained not only great developments but also inspirational accomplishments. However, the traditional traffic flow forecasting methods also involve some deficiencies, which make the method incapable of achieving a satisfying accuracy when forecasting traffic flows [1]. The deficiencies of the traditional traffic flow forecasting methods are reflected in the following four respects: firstly, the traditional traffic flow forecasting methods under Intelligent Transportation System only take into consideration traffic flow characteristics of current roads rather than impacts of traffic flows of adjacent roads on traffic flows of current roads. For example, when ARIM model is applied to forecast traffic flows, the method only considers traffic flow time series of current roads. Through exploration on rules of traffic flow time series, traffic flow forecasting is conducted. However, other factors are not considered, including upstream and downstream traffic flows. Therefore, the method cannot perfectly reflect traffic conditions of current roads when forecasting traffic flows, which makes accuracy improper. Secondly, some models view traffic conditions of upstream and downstream roads as a feature of model training, whereas these models leave aside influences of upstream roads on traffic flows of downstream roads, which make every influence an independent process [2]. For instance, suppose the downstream roads of road *A* include roads *B*, *C*, and *D*. When considering traffic flows of roads *B*, *C*, and *D*, it is obvious that the traffic flow of road *A* would influence them and for the traffic flow of road *A* would flow into them. But traditional traffic flow forecasting models take no account of effects of the merger. Thirdly, when updating models, traditional traffic flow forecasting methods should conduct another model training. Under this circumstance, large amounts of time and great computing resources spent are directly related to update frequency and accuracy of models. Particularly for methods under which modeling is troublesome, including Neural Network Algorithm, since their learning cycles are relatively long, these methods tend to update their methods infrequently. Fourthly, trajectory of crowds performs according to certain rules. In a specific period of time some traffic variables eventually approach steady values. Existing traffic flow forecasting methods, however, have not utilize constant values of traffic variables. So prediction training should reconsider these variables every time, which wastes considerable computing time and storage spaces [3]. For example, in a certain period of time, the transition probability of roads can be thought of as a constant value.  Table 1 shows features of the traditional traffic flow forecasting methods which we use in the paper in a comparative way.

In order to optimize accuracy of existing traffic flow forecasting methods, save time of training traffic flow forecasting models, and improve real-time capability of traffic flow forecasting, the thesis comes up with a Traffic Flow Forecasting Model Based on Transition Probability. The core concept of the model is, through analysis on historical traffic flow data, to yield transition probability values when upstream traffic flows transit to downstream traffic flows; then the value is combined with real-time traffic flows to forecast traffic flows. The method can dramatically cut time of model training and model update. Besides, the model considers impacts of upstream and downstream traffic flows on current traffic flows and relationship between a road and its upstream road, based on which traffic flow forecasting can achieve better performance [7].

The Traffic Flow Forecasting Model Based on Transition Probability includes the following main contents:

1. a modeling analysis on historical traffic flow data is conducted with the aim of obtaining transition probability of traffic flows of roads;
2. based on transition probability and real-time traffic flow data collected from real road networks, real-time traffic flow forecasting is carried out.

The algorithm should achieve the following goals:

1. to obtain transition probabilities of upstream traffic flow transiting to downstream traffic flow and ensure all values add up to 1;
2. to carry out momentary real-time traffic flow forecasting with time interval shorter than 5 minutes.

## 2. Related Work

Much of the research are related to traffic flow prediction. Abadi et al. have presented a work which accurately predicts short-term traffic flow rates up to 30 minutes ahead of time for an entire transportation network where traffic data are unavailable [8]. Lv et al. [9] propose a deep learning approach with a SAE model for traffic flow prediction, which can successfully discover the latent traffic flow feature representation. The work presented by Xing et al. [10] focuses on the decomposition of traffic flow matrix and introduced RPCA to accurately decompose the observation traffic flow matrix into submatrices that correspond to different classes of traffic flow.

In addition, Meng et al. [11] proposed using an online locally weighted learning method called LWPR for real-time traffic flow prediction, which does not need huge number of training data to build a global model in the beginning. Also, Tostes et al. [12] presented a methodology to obtain flow intensities from map services and use them to develop a prediction model which was designed to discover future flow intensities for a target street.

To sum up, those works mentioned above aim to solve the problem of traffic flow prediction. However, these works, to some extent, involve some deficiencies and can not solve the problem of traffic flow prediction perfectly. On the contrary, our approach differs from those works, and it can not only improve predicting accuracy but also reduce the training time. And we will explain it in detail later in this paper.

## 3. Brief Introduction for Model

**Figure 1:**
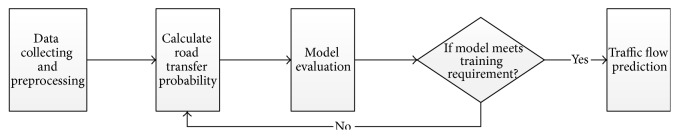
Flowchart of Traffic Flow Forecasting Based on Transition Probability.

Through analysis on historical traffic flow data, the Traffic Flow Forecasting Model Based on Transition Probability obtains transition probability of roads, based on which traffic flow is forecast. Forecast and modeling undergo the following five processes, as shown in Figure 1: first, collection and preprocessing of data; data here refer to records of traffic flows of roads and preprocessing includes structured process and stationary process of original data; second, computation of transition probability; this process mainly resorts to a constructed training set to compute transition probability through Newton Interior-Point Algorithm; third, performance evaluation of the model; the process mainly aims to evaluate whether the computed transition probabilities are in good agreement with true traffic flows; if not, training set should be readjusted and transition probability should be computed again; fourth, real-time traffic flow forecasting; traffic flows will be forecast according to the computed tradition probability.

## 4. Model by Transition Probability

**Figure 2:**
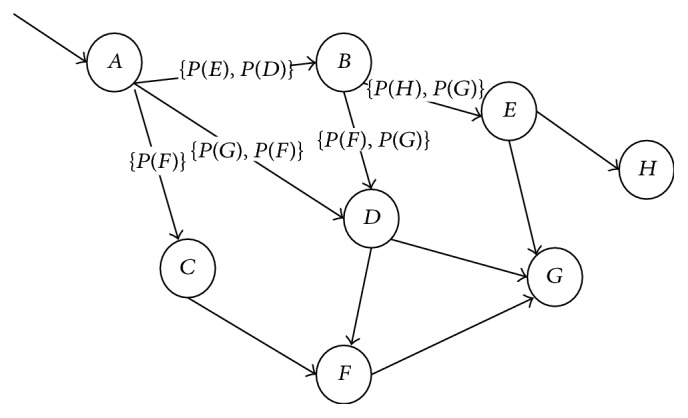
Schematic diagram of simulated road network.

The Traffic Flow Forecasting Model Based on Transition Probability is mainly designed to compute transition probability of roads, based on which traffic flows are predicted. When computing transition probability, road segments are chosen as the research object. Then the probabilities of vehicles in one road segment transiting to different downstream road segments shall be computed. In order to propose tradition probability computation theory more conveniently, a simulated road network is introduced as shown in Figure 2.

*A*, *B*, *C*,…, and *H* in Figure 2 serve as nodes in the road network. Lines between nodes represent road segments. Weight values beside road segments refer to probability values of transition to downstream road segments; for example, *P*(*E*) means probability of vehicles on *AB* road segment transiting to *BE* road segment. When forecasting traffic flows, suppose *F*(*AB*) represents traffic flows of *AB* road segment; *P*(*DG*) represents the transition probability of vehicles on *DB* road segment transiting to *DG* road segment; with *DG* road segment which is chosen as the research object, the traffic flow of *DG* road segment at the next moment is *F*(*BD*) × *P*_*BD*_(*DG*) + *F*(*AD*) × *P*_*AD*_(*DG*).

The Traffic Flow Forecasting Model Based on Transition Probability constructs its models based on consideration of relationship between real-time upstream and downstream traffic flows. Progresses of traffic flow forecasting can be shown by the following equation:

(1) yit+1=max⁡Xit+Qi,int−Qi,outt+Xdep,it−Xarr,it,0.

In the equation, *y*_*i*_(*t* + 1) represents predictive traffic flow of *i* road segment at *t* + 1 moment, *X*_*i*_(*t*) represents the traffic flow of *i* road segment at *t* moment, *Q*_*i*,in_(*t*) represents the traffic inflow of *i* road segment at *t* moment, *Q*_*i*,out_(*t*) represents the traffic outflow of *i* road segment at *t* moment, *X*_dep,*i*_(*t*) represents the number of vehicles starting from *i* road segment at *t* moment, and *X*_arr,*i*_(*t*) represents the number of vehicles stopping at *i* road segment at *t* moment. According to upstream traffic flow of *i* road segment and its corresponding transition probability, *Q*_*i*,in_(*t*) can be obtained:

(2) $$\begin{array}{c} Q_{i,in}\left(\;t\;\right)=\overset{u_{i}}{\underset{u=1}{\sum }}X_{\left(i-1\right)u}\left(\;t\;\right)\cdot P_{{\left(i-1\right)}_{u}\to i}\left(\;t\;\right)\cdot \alpha . \end{array}$$

In the equation, *X*_(*i* − 1)*u*_(*t*) represents traffic flow of *i* − 1, the upstream road segment of *i* road segment, at *t* moment, ·*P*_(*i* − 1)_*u*_→*i*_(*t*) represents transition probability of vehicles on *t* − 1 upstream road segment transiting to *i* road segment at *t* moment, and *u*_*i*_ represents an ensemble of all upstream road segments of *i* road segment.

*Q*_*i*,out_(*t*) can be obtained by three means, one of which is to acquire transition probability of vehicles on current road segments transiting to downstream road segments and traffic flows of current road segments according to the following equation:

(3) $$\begin{array}{c} Q_{i,out}\left(\;t\;\right)=\overset{d_{i}}{\underset{d=1}{\sum }}X_{i}\left(\;t\;\right)\cdot P_{i\to {\left(i+1\right)}_{d}}\left(\;t\;\right)\cdot \alpha . \end{array}$$

In the equation, *P*_*i*→(*i* + 1)_*d*__(*t*) represents transition probability of vehicles in *i* road segment transiting to *t* + 1 downstream road segment at *t* moment, and *d*_*i*_ represents an ensemble of all upstream road segments of *i* road segment.

Or it can be obtained by acquiring velocity and traffic density on current road segments according to the following equation:

(4) $$\begin{array}{c} Q_{i,out}\left(\;t\;\right)={\overset{-}{v}}_{i}\left(\;t\;\right)\cdot \frac{N_{i}\left(t\right)}{L_{i}}\cdot \Delta t. \end{array}$$

In the equation ${\overset{-}{v}}_{i}\left(t\right)$ represents the average speed of vehicles in *i* road segment at *t* moment, *L*_*i*_ represents the length of *i* road, *N*_*i*_(*t*) represents the amount of vehicles of *i* road, and Δ*t* represents time interval.

Or it can be obtained by computing the number of leaving vehicles based on ratio of average travel time and waiting time according to the following equation:

(5) $$\begin{array}{c} Q_{i,out}\left(\;t\;\right)=X_{i}\left(\;t\;\right)\times \frac{t}{T}. \end{array}$$

In the equation, *t* represents average travel time and *T* represents time interval.

When considering urban road networks, as roads have high mobility, the number of arriving vehicles and starting vehicles can be ignored. So the equation of changes of original traffic flows can be made up as follows:

(6) $$\begin{array}{c} y_{i}\left(\;t+1\;\right)=\max\left\{\;X_{i}\left(\;t\;\right)+Q_{i,in}\left(\;t\;\right)-Q_{i,out}\left(\;t\;\right),0\;\right\}. \end{array}$$

Through the above equation, solution of transition probability can be constructed. Then relevant solving algorithm can be applied to obtain final transition probability. Finally, traffic flows can be forecast.

**Figure 3:**
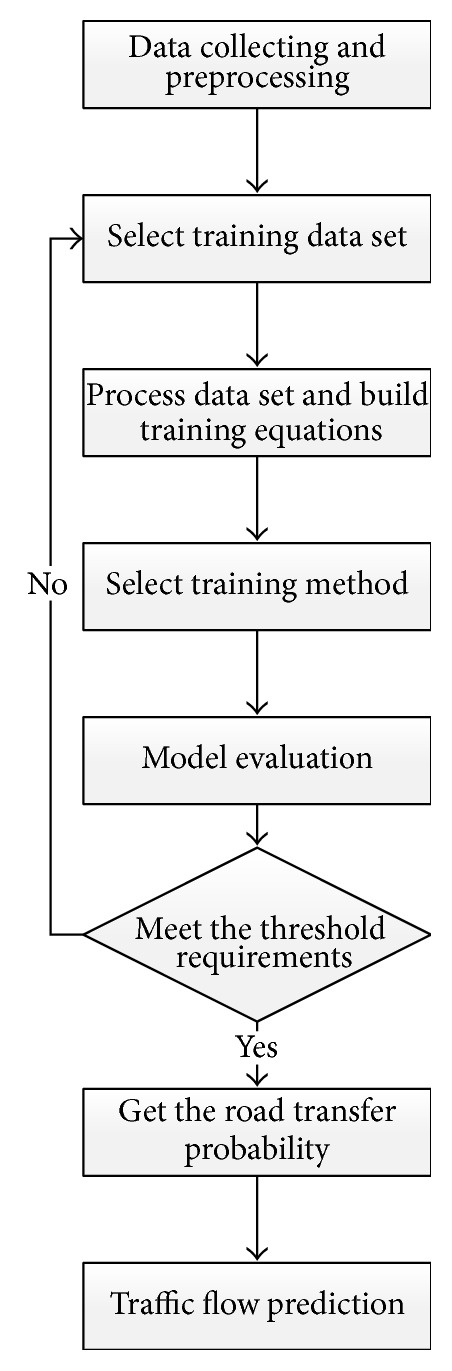
Flowchart of Traffic Flow Forecasting Based on Transition Probability.

The flowchart of Traffic Flow Forecasting Model Based on Transition Probability is shown in Figure 3.

When processing data set and constructing training equation module, lines and nodes in a road network can both act as a research object. When lines in a road network are chosen as the research object, the model can compute transition probability of vehicles on road segments transiting to different downstream road segments; when nodes are chosen as the research object, the model can construct training equations according to upstream lines related to selected node and then acquire transition probability of vehicles on upstream road segments related to the node transiting to different downstream road segments. If selected nodes serve as the research object, it is more convenient for training data processing and model training.

**Figure 4:**
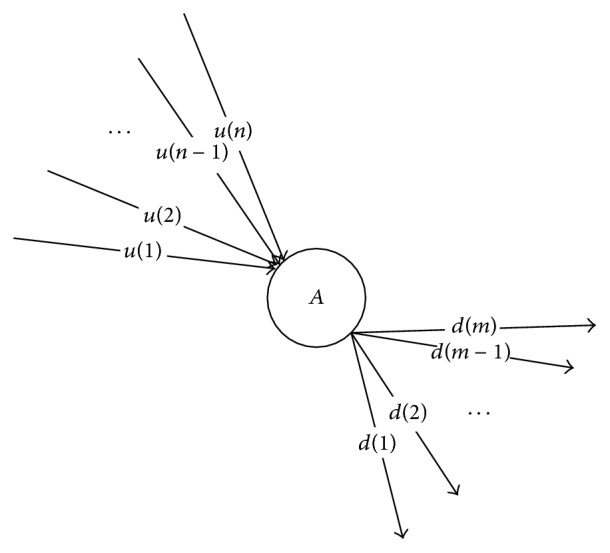
Schematic diagram of simulated node in road networks.

The following section introduces a simulated road network, in which *A* is the node of the road network. Besides, the ensemble of all upstream road segments of Node *A* is *U* = {*u*(1), *u*(2),…, *u*(*n*)} and the ensemble of all downstream road segments of Node *A* is *D* = {*d*(1), *d*(2),…, *d*(*m*)}. The road network is shown in Figure 4.

Suppose *X*_*i*_(*t*) represents traffic flows of *i* road segment in *t* moment, *y*_*i*_(*t*) represents predicative traffic flows of *i* road segment at *t* moment, and changes of original traffic can produce the equation

(7) ydit+1=Xdit+∑j=1UXujt·Puj→dit−Qdi,outtdi∈D

and through transformation can obtain

(8) ydit+1−Xdit+Qdi,outt=∑j=1UXujt·Puj→ditdi∈D.

Suppose ${\hat{y}}_{d\left(i\right)}\left(t\right)=y_{d\left(i\right)}\left(t+1\right)-X_{d\left(i\right)}\left(t\right)+Q_{d\left(i\right),out}\left(t\right)$,  ${\hat{X}}_{d\left(i\right)}\left(t\right)$ = [*X*_*u*(1)_(*t*), *X*_*u*(2)_(*t*),…, *X*_*u*(*n*)_(*t*)],  ${\hat{P}}_{U\to d\left(i\right)}\left(t\right)={\left[P_{u\left(1\right)\to d\left(i\right)}\left(t\right),P_{u\left(2\right)\to d\left(i\right)}\left(t\right),\ldots ,P_{u\left(n\right)\to d\left(i\right)}(t)\right]}^{T}$; the above equation can be reexpressed as

(9) $$\begin{array}{c} {\hat{y}}_{d\left(i\right)}\left(\;t\;\right)={\hat{X}}_{d\left(i\right)}\left(\;t\;\right)\cdot {\hat{P}}_{U\to d\left(i\right)}\left(\;t\;\right). \end{array}$$

If time frame selected, the following equation can be constructed:

(10) $$\begin{array}{c} Y_{d\left(i\right)}=X_{d\left(i\right)}\cdot P_{d\left(i\right)}. \end{array}$$

In the equation, $Y_{d\left(i\right)}=\{{\hat{y}}_{d\left(i\right)}\left(1\right),{\hat{y}}_{d\left(i\right)}\left(2\right),\ldots ,{\hat{y}}_{d\left(i\right)}\left(t\right)\}$,  $X_{d\left(i\right)}=\{{\hat{X}}_{d\left(i\right)}\left(1\right),{\hat{X}}_{d\left(i\right)}\left(2\right),\ldots ,{\hat{X}}_{d\left(i\right)}\left(t\right)\}$,  $P_{d(i)}=\{{\hat{P}}_{U\to d\left(i\right)}\left(1\right),{\hat{P}}_{U\to d\left(i\right)}\left(2\right),\ldots ,{\hat{P}}_{U\to d\left(i\right)}\left(t\right)\}$.

By solving the equation, transition probability of vehicles on upstream road segments transiting to downstream road segment $d(i)\;\;{\hat{P}}_{U\to d\left(i\right)}$ can be calculated. If equations of all downstream nodes of Node *A* are considered, the following equation group can be generated:

(11) $$\begin{array}{c} Y=X\times P, \end{array}$$

among which,

(12) Y=Yd1,Yd2,…,Ydm,X=Xd1⋯0⋮⋱⋮0⋯Xdm,P=Pd1,Pd2,…,PdmT.

Through training and solving the above equation, all transition probability values of upstream nodes related to Node *A* can be computed, based on which traffic flows of downstream road segment can be forecast.

## 5. Methods for Solving Model

Through a general introduction of the Traffic Flow Forecasting Model Based on Transition Probability, it is found that the model focuses on construction and solution of equation. The construction of equation is explained in the above chapter. Now this chapter mainly concentrates on solution methods of the equation. It is discovered from the construction of equation that the constructed equation is a linear equation. There are many ways to solve linear equations. And common solving methods for a linear equation include Ordinary Least Squares, Generalized Least Squares, Least Area Method, and Minimum Distance Method. Ordinary Least Squares is applied in the model.

### 5.1. A Linear Ordinary Least Squares

Ordinary Least Squares is the optimized technology by minimizing estimated squared errors. Ordinary Least Squares is applied to obtain values of variables and to minimize squared errors between predicated data and real data. Also known as OLS, Ordinary Least Squares is one of the most widely used parameter estimating methods.

The main problem Ordinary Least Squares should solve is that when observation data of samples have already acquired *Y* and *X*, the unclear mapping relation between *X* and *Y* is still to be found. Then OLS can be applied to obtain mapping equation and the most fitted sampled data based on the equation. In other words, the squared errors between estimated values and real values obtained by the mapping equation are minimal. The solution of OLS is operated as the following processes: suppose *Y* = {*y*(1), *y*(2),…, *y*(*n*)}, *X* = {*x*(1), *x*(2),…, *x*(*n*)} are known observation data. Then the mapping relation between *X* and *Y* is

(13) $$\begin{array}{c} Y=\beta \cdot X+b. \end{array}$$

When conducting solution by OLS, the object function is

(14) $$\begin{array}{c} Q=\overset{n}{\underset{i=1}{\sum }}{\left(\;y\left(\;i\;\right)-b-\beta x\left(\;i\;\right)\;\right)}^{2}. \end{array}$$

If values of object function are the minimal ones, it means that the mapping relation between *X* and *Y* is found. The reason why object function chooses sum of squares of difference between estimated values and real trues as its evaluation criterion is that simple sum can to a great extent offset great error values rather than revealing real estimation error and only sum of squares can overall reflect proximity between estimated values and real values, which is principle of OLS.

Since object function is a quadratic function of *b* and *β*, minimal values are bound to exist. Based on derivation of *Q*, when derivation is 0, object function *Q* reaches its minimal values. Then it can obtain

(15) ∂Q∂b=0,∂Q∂β=0.

The derivation of equations can give rise to

(16) β=∑i=1nxi−x−yi−y−∑i=1nxi−x−2,b=y−−βx−.

In the equation, $\overset{-}{x}=(1/n)\sum_{i=1}^{n}x_{i},\;\;\overset{-}{y}=(1/n)\sum_{i=1}^{n}y_{i}$.

The above equation is the most fundamental OLS concept. When applying OLS, the parameter estimation of the Traffic Flow Forecasting Model Based on Transition Probability is a process of multiple linear regression. Thus the equation can be written as

(17) $$\begin{array}{c} Y=X\beta . \end{array}$$

*Y* is the real value of training set while *X* is the character vector set among training set. Besides, *β* is the estimated parameter. Then the application of OLS concept can produce

(18) $$\begin{array}{c} \beta ={\left(\;X^{T}X\;\right)}^{-1}X^{T}Y. \end{array}$$

Through solution of the above equation, parameters of the model can be solved. However, in the Traffic Flow Forecasting Model Based on Transition Probability, the method is just a simple parameter estimation but the constraint condition of transition probability is not considered, which is all transition probability values in the Traffic Flow Forecasting Model Based on Transition Probability which should add up to 1.

### 5.2. Linear Ordinary Least Squares with an Inequality Constraint

As the application of Ordinary Least Squares for model solution cannot perfectly reflect requirements of transition probability values when forecasting traffic flows which is similar to common linear Ordinary Least Squares, with the aim of better reflecting practical significance of transition probability, Linear Ordinary Least Squares with an Inequality Constraint is applied to estimate model parameters.

Many researches have focused their attention on linear regression with an inequality constraint, which is an optimized problem. Its general concept is to transform questions into convex quadratic programming problems and then model parameters are solved. Through introduction of an inequality constraint of transition probability, the original equation can be transformed into

(19) Y=X×P,GP>W.

In the equation, *W* means constraint. According to transition probability which is bigger than 0 and smaller than 1, values of *G* and *W* are obtained, which are

(20) G=10⋯00−10⋯0101⋯000−1⋯01⋮⋮⋯⋮⋮00⋯1000⋯−11,W=0⋯0T.

*P* is a matrix of 1 × (*n∗m*), *G* is a matrix of 2*∗n∗m* × *n∗m*, and *W* is a matrix of 2*∗n∗m* × 1. Based on OLS concept, to solve the equation, the object function is

(21) min YTY−2YTXP+PTXTXPs.t. GP>W.

The above equation can be solved with the help of Optimization Theory, based on which the optimal solution can be achieved which meets the requirements of the inequality. Since *Y* ^*T*^ *Y* is a vector constant while *X* ^*T*^ *X* is a positively definite matrix, the equation strictly belongs to a convex quadratic programming problem and can be solved through solving its dual problem which is

(22) max ⁡YTY−2YTXP+PTXTXP−λTGP−W

(23) s.t. 2XTXP−2XTY−GTλ

(24)  λ≥0.

As known from Convex Quadratic Programming Theory, if $\tilde{P}$ and $\tilde{\lambda }$ are the optimal solution of the above equation, then $\tilde{P}$ is the optimal solution of the original equation. The above equation can lead to

(25) $$\begin{array}{c} \tilde{P}={\left(\;X^{T}X\;\right)}^{-1}X^{T}Y+\frac{1}{2}{\left(\;X^{T}X\;\right)}^{-1}G^{T}\tilde{\lambda }. \end{array}$$

When the above equation (22) is substituted into (21), it can obtain

(26) YTY−2YTXP+PTXTXP−λTGP−W=−14λTGXTX−1GTλ+λTW−GXTX−1XTY+YTY−YTXXTX−1XTY.

Thus, $\tilde{\lambda }$ is the optimal solution of the following programming problem:

(27) min 12λTDλ−λTC λ≥0

In the equation, *D* = (1/2)*G*(*X* ^*T*^ *X*)^−1^ *G* ^*T*^, *C* = *W* − *G*(*X* ^*T*^ *X*)^−1^ *X* ^*T*^ *Y*. In the equation, the optimal solution $\tilde{\lambda }$ of the above equation can be obtained through the utilization of Quadratic Programming Theory. To solve the above equation $\tilde{\lambda }$, Kuhn-Tucker condition should be meet, which is

(28) λ≥0,λTDλ−C=0,Dλ−C≥0.

An equation group can be acquired according to the above equation:

(29) $$\begin{array}{c} \lambda_{1}d_{i1}+\lambda_{2}d_{i2}+\cdots +\lambda_{m}d_{im}=c_{i}. \end{array}$$

**Figure 5:**
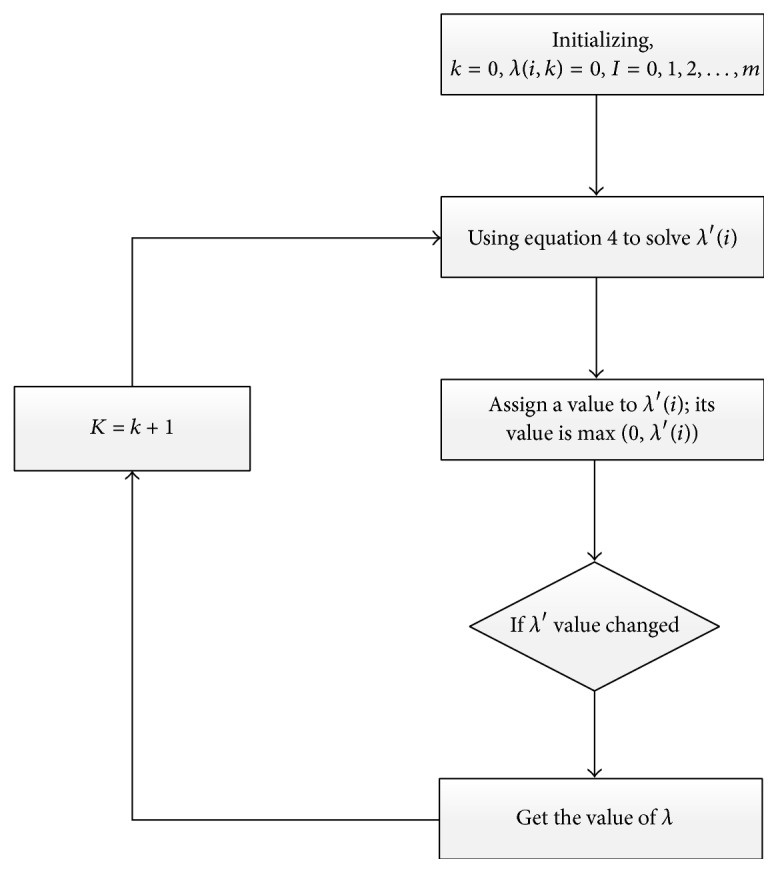
Flowchart of solution of the inequality constraint *λ*.

In the equation, *λ*_1_ is the element of *λ*, *d*_*i*1_ is the element of *D*, and *c*_*i*_ is the element of *C*. And the equation group $\tilde{\lambda }$ can be solved based on iteration. The solution processes are shown in Figure 5.

With the solution of $\tilde{\lambda }$, transition probability can be solved. The equation is

(30) $$\begin{array}{c} \tilde{P}={\left(\;X^{T}X\;\right)}^{-1}X^{T}Y+\frac{1}{2}{\left(\;X^{T}X\;\right)}^{-1}G^{T}\tilde{\lambda }. \end{array}$$

The Traffic Flow Forecasting Model Based on Transition Probability can take advantage of the solved transition probability value to forecast traffic flows.

### 5.3. Newton Interior-Point Algorithm

Though the application of Ordinary Least Squares with an Inequality Constraint to solve transition probability can meet requirements of transition probability in values, the method takes no account of relations of transition probabilities of one road. For one road, all vehicles head to downstream road segment. Thus its transition probabilities can add up to 1. To improve accuracy and authenticity of traffic flow forecasting, the constraint of all tradition probabilities of one road adding up to 1 should be introduced. With the introduction of equality constraints of transition probability, the original problem is transformed into a quadratic programming problem. Common methods applied to solve quadratic programming problems include Lagrange Method, Active Set Method, Interior-Point Algorithm, Infeasible Interior-Point Algorithm, and Branch-and Bound Method. The thesis applies Newton Interior-Point Algorithm to solve model parameters.

Through the introduction of equality constraints of transition probability, the original equation can be transformed into

(31) Y=X×P,GP>W,ATP=b.

In the equation, *A* and *b* are equality constraints. After *P* is constructed, the values of *A* and *b* can be obtained, which are

(32) A=10⋯001⋯0⋮⋮⋯⋮10⋯001⋯0⋮⋮⋯⋮00⋯1⋮⋮⋯⋮00⋯1,b=1⋯1T.

*A* is a matrix of (*n∗m*) × *n*, *b* is a matrix of *n* × 1, and elements in matrix *A* should be able to form the following relations:

(33) Am∗j+i,i=1i,j=1,2,…,n,Ai,j=0else.

For equality constraints which require transition probabilities of one road to add up to 1, the inequality constraints can be simplified as

(34) $$\begin{array}{c} P>0. \end{array}$$

With OLS concept applied, a constrained penalty function of linear regression is

(35) min YTY−2YTXP+PTXTXP.

Thus the original equation can be transformed into

(36) min QP=12PTGP+gTP, ATP=b, P>0.

In the equation, *G* = *X* ^*T*^ *X*, *g* = −*Y* ^*T*^ *X*.

With Logarithmic Penalty Function Method which applies, the original penalty function can be transformed into the following problems only with equality constraints:

(37) min BP,μk=12PTGP+gTP−μk∑i=1n∗mln Pi, ATP=b;

in the above equation, *μ*_*k*_ represents the parameter of the penalty function.

It is theoretically proven that values of *μ*_*k*_ under given condition can show the following trend: when *μ*_0_ > *μ*_1_ > *μ*_2_ > ⋯>*μ*_*∞*_ → 0 the original problem can produce the optimal solution.

Augmented Lagrangian Function can be applied to transform equality constrained optimization problems into unconstrained problems. The Augmented Lagrangian Function of the original problem is

(38) min LP,μk,v,σ=min⁡BP,μk−vTAP−b+σ2AP−bTAP−b.

In the equation, *σ* > 0, substituting min⁡*B*(*P*, *μ*_*k*_), can obtain

(39) min LP,μk,v,σ=12PTGP+gTP−μk∑i=1n∗mln Pi−vTAP−b+σ2AP−bTAP−b.

Then Newton's Method is applied to acquire the optimal solution. The iterative equation is

(40) Pk+1=Pk+λkdk,vk+1=vk−σXPk−b.

In the equation, *d* ^(*k*)^ is the optimized search direction and *λ*_*k*_ is the step of iteration. And the value of *d* ^(*k*)^ can be solved based on the following equation:

(41) $$\begin{array}{c} \nabla_{P}^{2}L\left(\;P,v\;\right)\Delta d=-\nabla_{P}L\left(\;P,v\;\right), \end{array}$$

among which

(42) ∇PLP,v=g+GP−μX−1e−Av+σAAP−b,∇P2LP,v=G+μX−2+σAA′.

Here *X* = Diag(*p*_1_, *p*_2_,…, *p*_*n*_), *e* = (1,1,…,1)^*T*^ ∈ *R* ^*n*^.

To ensure feasibility of solutions, appropriate *λ*_*k*_ should be chosen. The value of *λ*_*k*_ can be acquired based on the following equation:

(43) $$\begin{array}{c} \lambda_{k}=\gamma \min\left\{\;1,-\frac{p_{k}^{j}}{d_{k}^{j}}\;|\;d_{k}^{j}<0\;\right\}. \end{array}$$

The value of *γ* should range from 0 to 1 and generally it is 0.9995.

**Figure 6:**
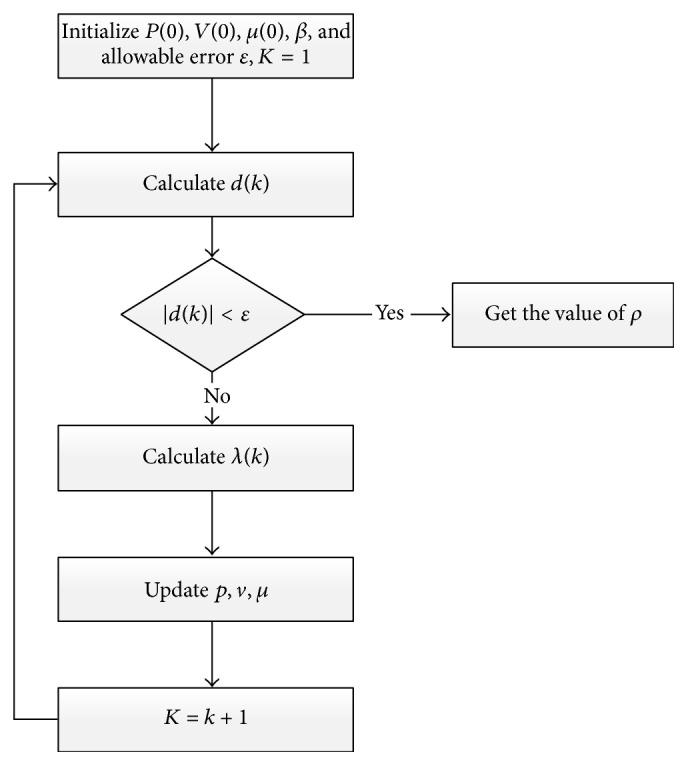
Flowchart of implementation of Newton Interior-Point Algorithm.

Procedures of algorithm implementation are shown in Figure 6.

The implementation of Newton Interior-Point Algorithm can solve for the probability value of constrained Traffic Flow Forecasting Model Based on Transition Probability, based on which traffic flows are to be forecast.

## 6. Experiment

### 6.1. Evaluation Criteria

To evaluate performances of traffic flow forecasting, the paper adopts MAE, MAPE, and RMSE as evaluation criteria for performances of the model. MAE refers to Mean Absolute Error, reflecting expected values of prediction error [13]. The smaller the MAE is, the more accurate the traffic flow forecasting is. Its solution equation is

(44) $$\begin{array}{c} MAE=\frac{1}{T}\overset{T}{\underset{t=1}{\sum }}\left|\;\hat{X}\left(\;t\;\right)-X\left(\;t\;\right)\;\right|. \end{array}$$

In the equation, $\hat{X}\left(t\right)$ represents predicative values while *X*(*t*) represents true values. MAPE is mean absolute percentage error and its solution equation is

(45) $$\begin{array}{c} MAPE=\frac{1}{T}\overset{T}{\underset{t=1}{\sum }}\frac{\left|\hat{X}\left(t\right)-X\left(t\right)\right|}{X\left(t\right)}\times 100\%. \end{array}$$

RMSE is Root Mean Square Error. With its ability to well reflect accuracy of forecasting, RMSE is widely applied in engineering measurement. Its solution equation is

(46) $$\begin{array}{c} RMSE=\sqrt{\frac{1}{T}\overset{T}{\underset{t=1}{\sum }}{\left|\hat{X}\left(t\right)-X\left(t\right)\right|}^{2}}. \end{array}$$

The paper simultaneously applies the three evaluation criteria to reflect accuracy of traffic flow forecasting.

### 6.2. Experiment Environment

**Figure 7:**
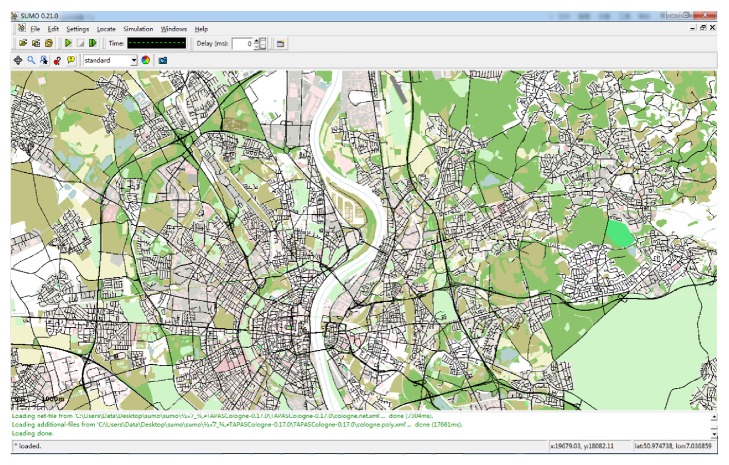
SUMO interface.

SUMO platform, developed by German Aerospace Center, is a micro- and continuous simulated architecture of road traffic and a model basis. SUMO platform enables its users to load different road networks and set various traffic streams. Therefore it is extremely suitable for researchers who focus on road traffic simulation researches. SUMO is a platform with open sources, programmed in c++ language. Besides, it provides graphical interfaces just like Figure 7.

### 6.3. Experimental Parameters

Combining traffic conditions of urban road networks, the paper explores traffic flow forecasting which falls into the category of momentary traffic flow forecasting. In transportation systems, traffic speed, traffic volume, and traffic density are basic features of traffic flow. And acquisition of accurate traffic variables is of great practical significance for analyzing and modeling of traffic flows as well as detection of traffic emergencies and accidents. Traffic flow forecasting is currently the most heated research object and relatively mature traffic flow parameter forecasting. Besides, many ways to forecast traffic flows have been invented.

Momentary traffic flow forecasting requires that the time frames of forecasting should be no longer than 15 minutes which is still a long duration for urban road networks whose road segments are shorter. So the mean travel time in the shortest road segment of a road network should be set as the time frame of traffic flow forecasting, which requires efficiency of forecasting to be high. With time frame of forecasting shortening, traffic flow forecasting can be troubled with more interference factors. And sometimes the statistical behaviors can be uncertain and completely random. Under this circumstance, the time frame is not suitable for forecasting. Thus the time frame should be lengthened or other methods should be applied to describe traffic flows [14].

**Figure 8:**
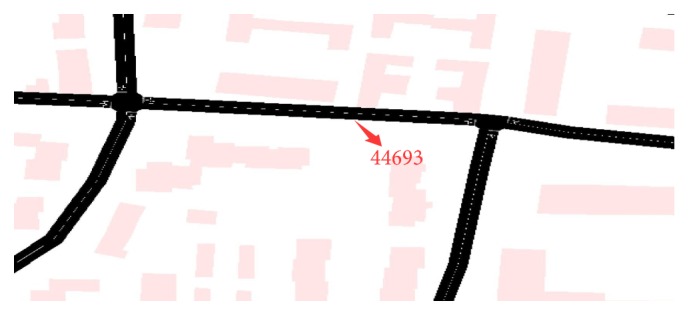
Road map of road segment number 44693.

Since urban road networks are strongly dynamic, the paper adopts various time frames to collect traffic flow data, which are 10 s, 30 s, 60 s, 120 s, 180 s, 240 s, and 300 s. And based on analysis on the collected traffic flow data, a suitable time frame is determined. Then this time frame is used for traffic flow forecasting. Besides, the traffic flow data that are integrated through a simulator can more effectively analyze traffic flows. Afterwards, historical traffic flow data are analyzed with the purpose of forecasting the traffic flow in the next moment, through which traffic participants can choose the optimal driving route. Since traffic conditions of a whole road network can be too complicated, the thesis focuses on one road segment of a road network to carry out traffic flow forecasting, with road segment number 44693 chosen as the research object. The road network of road segment number 44693 is shown in Figure 8.

One minute is chosen as the time frame of data collection in the experiment. The following section applies Time Series Analysis, Kalman Filter Forecasting Method, Neural Network Forecasting Method, and Traffic Flow Forecasting Method Based on Transition Probability to forecast and analyze traffic flows of road segment number 44693.

### 6.4. Comparison of Multiple Models

#### 6.4.1. Time Series Model for Traffic Flow Forecasting

When implemented, before forecasting traffic flows, Time Series Model for Traffic Flow Forecasting will set model parameters based on traffic flow data collected in a 10-second time frame. The training mode and predictive model are shown in Table 2 [15].

In the experiment, parameters of ARIMA(*p*, *d*, *q*) are, respectively, *p* = 18, *d* = 1 and *q* = 0. Then traffic flow forecasting can undergo with the support of the model.

We found that the forecasting performance of Time Series Analysis is not very satisfying, with time delay involved. Thus the model cannot produce excellent forecasting of traffic flows in future periods of time. Besides, historical traffic flow data have to be collected repeatedly in training process. And model calculations must be conducted again whenever traffic flows in future moments are to be forecast, which decreases forecasting efficiency. However, it is also found that when forecasting traffic flows based on Time Series Analysis, its predicted traffic flow trend is the same as the original one. As a result, it can be used to predict traffic flow trend. If time series are applied to forecast traffic flows, MAPE is 43.32; MAE is 1.24; RMSE is 1.54; and predicated number in the experiment is 50. Besides, algorithm is high sensitive to data, making forecasting undulate. Therefore, when time series is utilized for traffic flow forecasting, data should be preprocessed.  Table 3 shows the result of Time Series Model Traffic Flow Forecasting with different parameters.

#### 6.4.2. Kalman Filtering Traffic Flow Forecasting

When Kalman Filtering Algorithm is applied in traffic flow forecasting, initial values of model training parameters should be determined and features of training set should be constructed. Then Kalman Filtering Traffic Flow Forecasting should be utilized to develop model parameters [16]. The training mode and predicative model are shown in Table 4.

In the experiment, 6 characteristics are chosen as inputs of the model. Suppose that *v*(*d*, *t*) represents the traffic flow in *t* moment of the *d* day. So training characteristics are *v*(*d*, *t*), *v*(*d*, *t* − 1), *v*(*d* − 1, *t*), *v*(*d* − 1, *t* − 1), *v*(*d* − 2, *t*), and *v*(*d* − 2, *t* − 1). Then *X*(0) and *P*(0) are set as 0 vectors. And the input is used for parameter estimation through Kalman Filtering Algorithm [17]. Finally traffic flows are forecast.

Through the experiment, it is found that the forecasting results of Kalman Filtering Algorithm are not very satisfying. The results are subject to undulation and low accuracy. However, modeling is fast and leads to satisfying model parameters. In the experiment, performance indicators created by Kalman Filtering Algorithm include MAPE which are 83.23%, MAE 2.25, and RMSE 3.18. And it forecasts traffic flows 50 times.  Table 5 shows the result of Kalman Filtering Traffic Flow Forecasting with different parameters.

#### 6.4.3. Neural Network Traffic Flow Forecasting

If BP neural network is used to forecast traffic flows, number of input units, hidden units, and output units should be determined at first. Then training times and training objective values of training should be determined. Finally BP neural network is applied in training model parameters [18]. The training mode and predicative model of BP neural network are shown in Table 6.

In the experiment, 5 characteristics are chosen as inputs of the model. Suppose *x*(*t*) means traffic flows at *t* moment. Then training characteristics are *x*(*t*), *x*(*t* − 1), *x*(*t* − 2),…, *x*(*t* − *p*). Length *p* is set as 5 and output value is *x*(*t* + 1), namely, *y*(*t*). During model training of BP network, training time is set as 15000, with target set as IE-6. And the number of input units is 5; the number of output units is 1; and the number of hidden units is 5. Besides, transition functions are all log-sigmoid. Based on this input, model training of BP neural network and traffic flow forecasting are successively carried out.

We found that BP neural network is equipped with excellent stability in forecasting and high degree of fitting. However, the forecast of unknown data is relatively low in accuracy. Besides, the modeling is time-consuming. And the network is unable to adapt to unexpected traffic flows. In the experiment, performance indicators obtained by BP neural network include MAPE which is 41.45%, MAE 0.97, and RMSE 1.51. And the network forecasts traffic flows 50 times.  Table 7 shows the result of Neural Network Traffic Flow Forecasting with different parameters.

#### 6.4.4. Traffic Flow Forecasting Method Based on Transition Probability

If Traffic Flow Forecasting Method Based on Transition Probability is applied, firstly road nodes should be selected to construct training equations during which time frames of training set should be determined because transition probabilities in different time of one day are different. Different road segments can have the same time frame. Then a model training method should be determined. Model training methods include Ordinary Least Squares and Linear Ordinary Least Squares with an Inequality Constraint and Newton Interior-Point Algorithm. After the method is applied, transition probability can be solved and stored into a database for further traffic flow forecasting; when forecasting traffic flows, only transition probability value of upstream road segments of appointed road in the current moment should be obtained. Then the value should be combined with real-time traffic volume to work out the traffic flow in the next moment. The training mode and predicative model of Traffic Flow Forecasting Method Based on Transition Probability are shown in Table 8.

As indicated in Table 8, *n* means the number of upstream road segments of current node; *x*_*i*_(*t*) represents the number of vehicles on *i* road segment at *t* moment; input *y*(*t*) refers to input of road segments to be forecast at *t* moment.

The experiment chooses 44693 road segment as its node. And the transition relation between relevant upstream and downstream road segments is shown in Table 9.

Through the construction of training set, transition probability is solved based on which traffic flow can be forecast.

The experiment utilized three methods to solve model parameters, including Ordinary Least Squares, Least Squares with Inequality Constraints, and Newton Interior-Point Algorithm. The parameter values calculated by the three methods are shown in Table 10.

**Figure 9:**
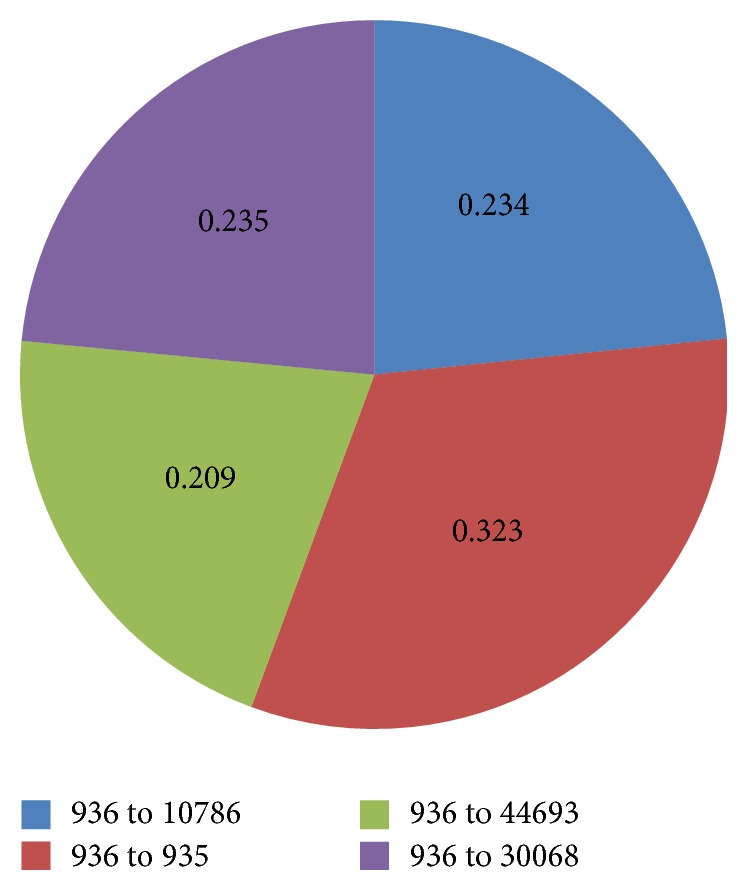
Distribution of transition probabilities.

**Figure 10:**
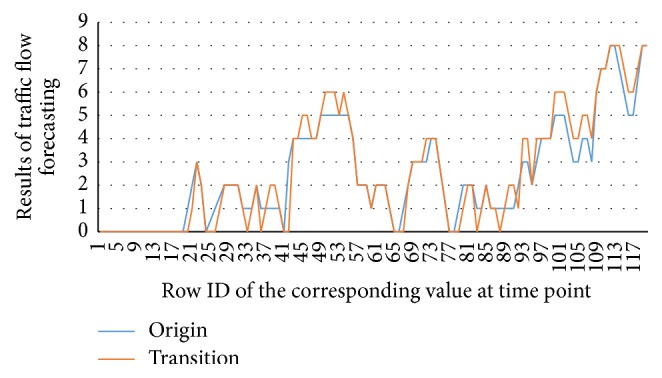
Results of Traffic Flow Forecasting Based on Transition Probability.

Table 11 indicates that model parameter values computed by OLS do not match with actual probability values. Besides, those parameters computed by Least Squares with Inequality Constraints, though range from 0 to 1, are mainly closer to minimal probability values given by the system. Thus those values cannot excellently reflect conditions of traffic flows. Parameter values calculated by Newton Interior-Point Algorithm range from 0 to 1. And transition probability values of one upstream road segment add up to 1, meeting requirements of actual probability values. And it is found that Newton Interior-Point Algorithm is advantageous in accuracy. The following part analyzes parameter values and predicative results of Newton Interior-Point Algorithm.  Figure 9 shows distribution of transition probabilities, and Figure 10 shows the results of Traffic Flow Forecasting Based on Transition Probability.

The experiment shows that the model is relatively steady and accurate, identical to actual traffic flows. When solving transition probability, the experiment shows that upstream road segments of target road include 936 road segment, 10787 road segment, 30067 road segment, and 44692 road segment. According to the transition probability of 936 road segment shown in Figure 10, transition probabilities add up to 1, every one ranging from 0 to 1, which meets the requirement of transition probability of actual traffic. The results show the accuracy of Traffic Flow Forecasting Based on Transition Probability. In addition, the forecasting results are also satisfying. In the experiment, performance indicators obtained by Traffic Flow Forecasting Based on Transition Probability include MAPE which are 30.3%, MAE 0.758, and RMSE 1.13. And it forecasts traffic flows 50 times.

## 7. Evaluation of the Result of Experiment

**Figure 11:**
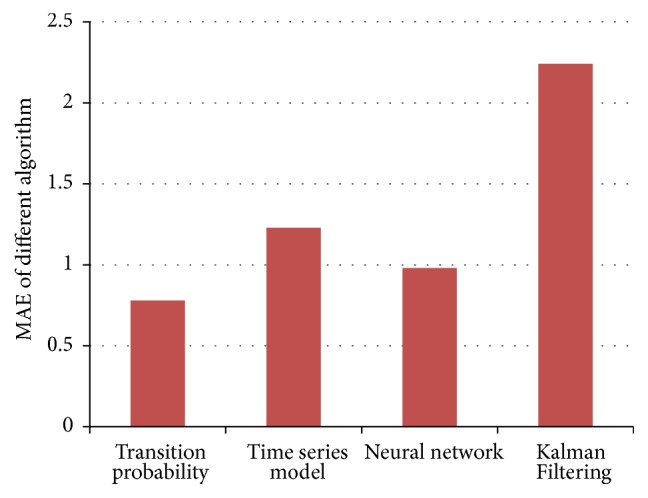
MAE indicator of Every Forecasting Algorithm.

**Figure 12:**
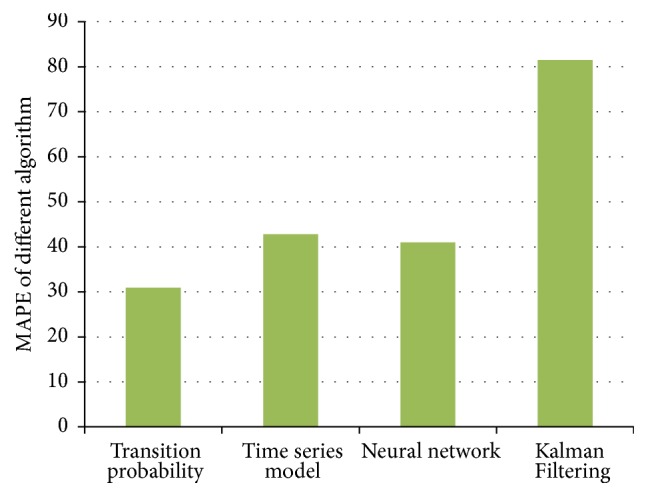
MAPE indicator of Every Forecasting Algorithm.

**Figure 13:**
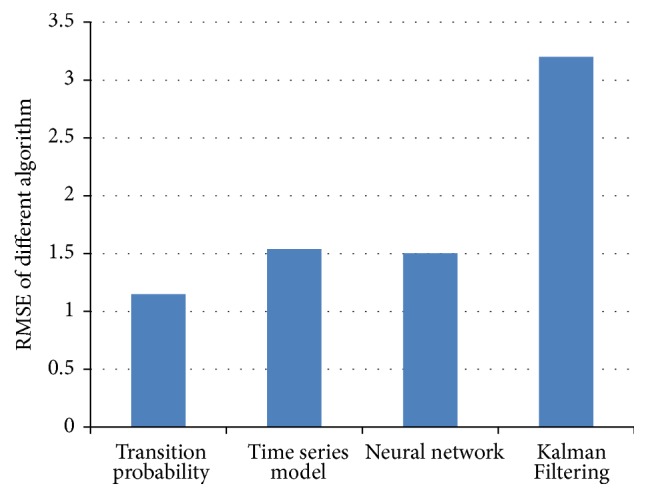
RMSE indicator of Every Forecasting Algorithm.

**Figure 14:**
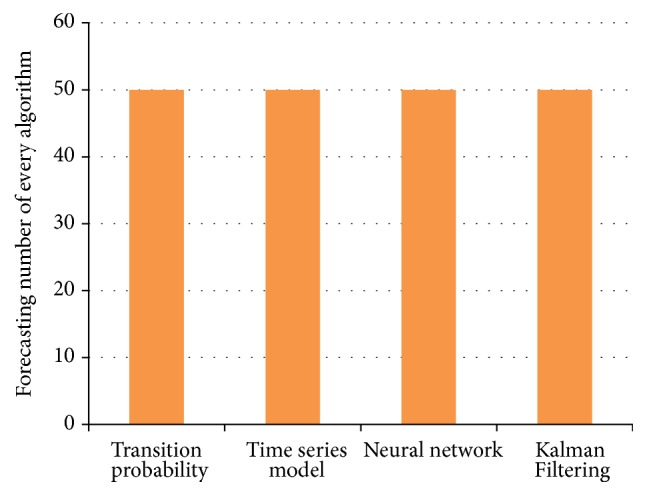
Forecasting number of every algorithm.

**Figure 15:**
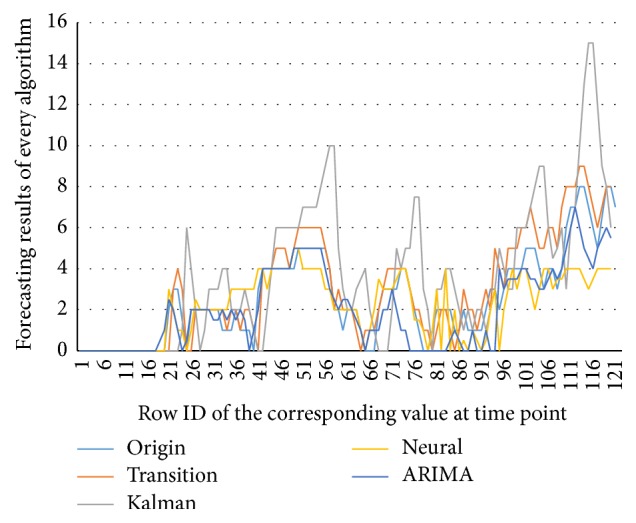
Traffic flow forecasting results of every algorithm.

The thesis, respectively, applies ARIMA Analysis, Kalman Filtering Algorithm, BP Neural Network Algorithm, and Traffic Flow Forecasting Based on Transition Probability to predicatively analyze traffic flows of Cologne. Besides, the paper also analyzes experimental results of these algorithms. In addition, a comparative analysis on these algorithms is conducted. The experimental results of these algorithms are shown in Figures 11, 12, 13, 14, and 15: Figure 11 shows the value of MAE; Figure 12 shows the value of MAPE; Figure 13 shows the value of RMSE; Figure 14 shows forecasting number of every algorithm; Figure 15 shows traffic flow forecasting results of every algorithm.

Judging from the experimental results shown in Figure 15 and results shown in figures of performance indicators of every algorithm, Traffic Flow Forecasting Based on Transition Probability is more accurate, followed by Neural Network Algorithm, Time Series Analysis, and Kalman Filtering Algorithm. Besides, analysis on the experimental results can find that these algorithms have their own characteristics.

Firstly, as for forecasting accuracy, Neural Network Algorithm and Traffic Flow Forecasting Based on Transition Probability are similarly accurate. But the training process of Neural Network Algorithm is more complicated and poor in adaptability to unexpected circumstances in road networks. And Traffic Flow Forecasting Based on Transition Probability is endowed with more convenient modeling and solving processes. However, since Traffic Flow Forecasting Based on Transition Probability takes into consideration impacts of upstream and downstream road segments on traffic flows of current road segment, which makes the method more adaptable to contingencies taking place in roads, Traffic Flow Forecasting Based on Transition Probability is more suitable for real-time traffic flow forecasting, whereas Kalman Filtering Algorithm and Time Series Analysis are less accurate. Besides, Kalman Filtering Algorithm influences to a great extent setting of initial values.

Secondly, as for robustness of models, Traffic Flow Forecasting Based on Time Series and Kalman Filtering Algorithm present great undulations when forecasting traffic flows while Neural Network Algorithm and Traffic Flow Forecasting Based on Transition Probability are relatively steady and able to conduct better traffic flows forecasting.

Thirdly, as for efficiency of modeling, Traffic Flow Forecasting Based on Transition Probability takes the shortest time in modeling and solving, followed by Kalman Filtering Algorithm and Time Series Analysis. However, Neural Network Algorithm takes longer time for model training, since its model training needs to repeatedly use its training set to regulate model parameters, which is a iterative process. Thus Neural Network Algorithm is defective in model update and real-time forecasting.

Fourthly, as for complexity of model parameters, Traffic Flow Forecasting Based on Transition Probability and Kalman Filtering Algorithm have relatively simple parameters, advantageous in model storage. Besides, as training sets increase, the parameter update of Kalman Filtering Algorithm, which is completed through iterative operations based on the original model, is simpler and more convenient to implement. And the parameter update of Traffic Flow Forecasting Based on Transition Probability, relatively simple as well, can be completed by reselection of a training set, based on which parameters and new transition probability can be solved.

Characteristics of all these algorithms are embedded in the experimental results. Hereby characteristics of these algorithms are concluded as shown in Table 12.

Through analysis on these traffic flow forecasting models, it is found that these models all involve shortages. Thus forecasting models can be combined; thus advantages of the models can be utilized at utmost so as to improve accuracy and real-time capability of traffic flow forecasting. Combined forecasting models mainly exist in the following three forms: the first is to adopt different forecasting method according to different time frames; the second is to regulate the number of combined forecasting algorithms as time changes; the third is to regulate weights of models as time changes and view weighted values as final forecasting values. So Time Series Analysis, Kalman Filtering Algorithm, Neural Network Algorithm, and Traffic Flow Forecasting Based on Transition Probability can be combined according to the third form.

## 8. Conclusion

The thesis makes use of Cologne's true traffic flow data to analyze advantages and disadvantages of some traffic flow forecasting algorithms. On this basis, the thesis comes up with Traffic Flow Forecasting Based on Transition Probability. Our contribution in this paper is threefold.

1. Analysis and verification of existing traffic flow algorithms and exploration of applicability of these algorithms: this paper mainly adopts several existing traffic flow forecasting algorithms, including Traffic Flow Forecasting Based on Time Series, Kalman Filtering Algorithm, and BP Neural Network Forecasting Algorithm.
2. Proposal of Traffic Flow Forecasting Based on Transition Probability: the method considers not only traffic conditions of current road segments but also traffic flow conditions of adjacent road segments. Through computation of transition probabilities in different moments, traffic flows are forecast. Besides, the method also conducts an experiment that discovers the algorithm which can satisfy demands of momentary traffic flow forecasting. Besides, its computation time is short, which enables the algorithm to be suitable for real-time momentary traffic flow forecasting. In addition, the method also introduces inequality constraints of transition probability and an equality constraint which requires all transition probabilities to add up to 1, allowing the algorithm to better adapt to real traffic flow forecasting environments.
3. Analysis on characteristics of traffic flows of urban road segments based on which inputs and outputs of all algorithms are constructed.

The paper analyzes Ordinary Least Squares and Ordinary Least Squares with Inequality Constraints and Newton Interior-Point Method. Then these methods are applied to obtain model parameters and perfectly solve problems of model parameter estimation.

Real-time momentary traffic flow forecasting of urban road networks is of great complexity. The reasons are that traffic conditions of urban road networks are time-varying and more dynamic than those of highways and that factors that influence traffic flows are various. The paper still suffers from some limitations, which are main research contents in the future.

1. According to traffic conditions of urban road networks, to automatically recognize time frames during which traffic flows conform to the same rules rather than to set time frames artificially: in this way, traffic flows can be forecast in a more intelligent way and adaptability of models can be improved.
2. According to different road segment, to adopt different forecasting time frames: it is known from analysis on traffic flows that some road segments have fast changing traffic flows while some have slowly changing traffic flows. For road segments with slowly changing traffic flows, forecasting time frames should be longer and vice versa. In this case, forecasting can be more effective, efficient, and accurate.
3. To improve model solution methods: as for estimating parameters that involve inequality and equality, parameter estimation is inefficient. Thus algorithms should be developed so as to improve efficiency of parameter estimation. In this way, algorithms can be provided with stronger real-time capability and traffic flow forecasting speed can be life.

Traffic flow forecasting models all reflect their own merits and demerits. Meanwhile, currently, the accuracy of traffic flow forecasting is unsatisfying, especially of traffic flow forecasting models for urban road networks. So merits of models should be fully taken advantage of and models should be integrated so as to improve accuracy of traffic flow forecasting.

## Figures and Tables

**Table 1:** Methods features.

Methods	Advantages	Disadvantages	For example
Times series	The forecast trend is practical	Time delay and time-consuming	Paper [4]
Kalman Filtering	It is fast and satisfies model parameters	The forecast trend is not practical	Paper [5]
BP neural network	Stability in forecasting and high degree of fitting	Time-consuming and low accuracy in prediction	Paper [6]

**Table 2:** Input and output of Time Series Model for Traffic Flow Forecasting.

Training mode	Input	(1) ID road segment (2) Time frame (3) Parameters of set model training: *p*, *d*, and *q* (4) Training data: arrange traffic flows of current road segment in a time series
	Output	Traffic flows of the road segment in the next moment, model parameters

Predicative mode	Input	Model parameters, time series data
	Output	Traffic flows in the next moment

**Table 3:** The result of Time Series Model Traffic Flow Forecasting with different parameters.

*ε*	MAE	MAPE	RMSE
1.00*E* − 02	0.616666666666666	25.8174603174603	1.05672449894315
1.00*E* − 03	0.641666666666666	26.5119047619047	1.06066017177982
1.00*E* − 04	0.7	28.3035714285714	1.09544511501033
1.00*E* − 05	0.708333333333333	28.4285714285714	1.10679718105893
1.00*E* − 06	0.708333333333333	28.4285714285714	1.10679718105893
1.00*E* − 07	0.708333333333333	28.4285714285714	1.10679718105893

**Table 4:** Inputs and outputs of Kalman Filtering Traffic Flow Forecasting.

Training mode	Input	(1) ID road segment (2) Time frame (3) Determination of training characteristics (4) Initialized model parameters
	Output	model parameters

Predicative mode	Input	Model parameters. Characteristic values of road segments in current moments
	Output	Traffic volume in the next moment

**Table 5:** The result of Kalman Filtering Traffic Flow Forecasting with different parameters.

Input	LAMDA	MAE	MAPE	RMSE
4	10	3.47	88.97	4.18
4	12	3.45	88.69	4.14
4	8	3.45	88.69	4.14
3	10	1.25	36.42	1.71
3	7	1.43	42.56	1.87

**Table 6:** Inputs and outputs of Neural Network Traffic Flow Forecasting.

Training mode	Input	(1) ID road segment (every road segment conforms to different rules, so training parameters can be different as well) (2) Time frame (time period in one day) (3) Time frame of training set (data collected in different days) (4) Determination of training characteristic (5) Initialized model parameter
	Output	Parameters of neural network model

Predicative mode	Input	Model parameters, characteristic values of the road segment in current moment
	Output	Traffic flow in the next moment

**Table 7:** The result Neural Network Traffic Flow Forecasting with different parameters.

Input	Hidden	Output	Count	MAE	MAPE	RMSE
4	5	1	100000	1.63	43.19	2.14
5	4	1	100000	1.51	39.03	2.08
3	3	1	100000	1.39	30.99	1.98
3	4	1	100000	1.29	29.04	1.88
4	3	1	100000	1.57	36.09	2.19

**Table 8:** Inputs and outputs of Traffic Flow Forecasting Method Based on Transition Probability.

Training mode	Input	(1) node ID, used to determine upstream and downstream road segments (2) Time frame (time period in one day) (3) Time frame of training set (data collected in different days) (4) Initialize training set: *X* = [*x*_*ij*_(*t*)]; *Y* = {*y*_*ij*_(*t*)}; *P* = {*p*_*ij*_(*t*)}
	Output	Transition probability of vehicles on upstream road transiting to downstream road based on the current node, *p*

Predicative mode	Input	〈*x*_1_(*t*), *x*_2_(*t*), *x*_3_(*t*),…, *x*_*n*_(*t*)〉 〈*p*_1_(*t*), *p*_2_(*t*),…, *p*_*n*_(*t*)〉
	Output	*y*(*t* + 1) traffic flow in the next moment

**Table 9:** Transition relation between relevant road segments.

936	10786
936	935
936	44693
936	30068
10787	30068
10787	10786
10787	935
10787	44693
30067	44693
30067	30068
30067	10786
30067	935
44692	935
44692	44693
44692	30068
44692	10786

**Table 10:** Transition probability values computed by different algorithm.

	Ordinary Least Squares	Least Squares with Inequality Constraints	Newton Interior-Point Algorithm
936 → 10786	−0.02925	0.05	0.233709
936 → 935	0.13821	0.142788	0.322543
936 → 44693	−0.19347	0.05	0.208581
936 → 30068	−0.02687	0.05	0.23508
10787 → 30068	−0.1021	0.05	0.345658
10787 → 10786	−0.03057	0.05	0.49872
10787 → 935	−0.2355	0.05	0.155605
10787 → 44693	−0.30255	0.05	0.05
30067 → 44693	0.314872	0.188902	0.337673
30067 → 30068	0.036905	0.05	0.205906
30067 → 10786	−0.01411	0.05	0.205237
30067 → 935	0.043521	0.05	0.250905
44692 → 935	−0.25781	0.05	0.20343
44692 → 44693	0.356332	0.146524	0.412875
44692 → 30068	0.104941	0.058461	0.245696
44692 → 10786	−0.02311	0.05	0.137982

**Table 11:** Parameter predication performances of different algorithm.

	Ordinary Least Squares	Least Squares with Inequality Constraints	Newton Interior-Point Algorithm
MAE	0.823529412	0.795098039	0.758333333
MAPE	30.25676937	31.00186741	30.3234127
RMSE	1.084652289	1.06642154	1.136515141

**Table 12:** Comparison on characteristics of algorithms.

Algorithm	Characteristics
Kalman Filtering Algorithm	The model's structure is relatively flexible and its solution process is simple and easy to implement. It is strongly adaptive. However, the initial values of the model are hard to be determined, which can influence forecasting effects. How to find a more suitable initial values is a direction for improvement of Kalman Filtering Algorithm

Neural Network Algorithm	The model is accurate in forecasting and steady as well. But its training speed is relatively slow. Besides, the setting of initial values that are connected to weight values in the neural network can directly influence speed of neural network training and eventual forecasting accuracy. In addition, the model cannot be popularized. Every roadway adopts its own model, so the model management is not very convenient. Thus how to improve the speed of network training is a direction of improvement of Neural Network Algorithm and also a demand of real-time traffic flow forecasting

Traffic Flow Forecasting Based on Time Series	The algorithm can carry out real-time forecasting. However, the model has many shortages. For example, the model has strict requirements for training data sets. Or its forecasting accuracy can be influenced greatly. Researches on time series provide many ways to improve the forecasting efficiency of the algorithm

Traffic Flow Forecasting Based on Transition Probability	The model is not complicated in computation and is able to quickly obtain model parameters. In addition, its forecasting accuracy is similar to the forecasting accuracy of Neural Network Algorithm; thus the model is suitable for forecasting real-time traffic flows. Meanwhile, Traffic Flow Forecasting Based on Transition Probability is easy to update and does not strictly require data to be in time series. Besides, the method is highly adaptive and flexible. However, the modeling and solution of the method are relatively complicated. So how to improve implementation of modeling and construction of training sets is a further direction for improvement
